# Characterization of *Pf*TrxR inhibitors using antimalarial assays and *in silico* techniques

**DOI:** 10.1186/1752-153X-7-175

**Published:** 2013-11-10

**Authors:** Ranjith Munigunti, Symon Gathiaka, Orlando Acevedo, Rajnish Sahu, Babu Tekwani, Angela I Calderón

**Affiliations:** 1 Department of Pharmacal Sciences, 4306 Walker Building, Auburn University, Auburn, AL, USA; 2 Department of Chemistry and Biochemistry, Auburn University, Auburn, AL, USA; 3 National Center for Natural Products Research & Department of Pharmacology, Research Institute of Pharmaceutical Sciences, School of Pharmacy, University of Mississippi, University, MS, USA

**Keywords:** Malaria, *Plasmodium falciparum*, Thioredoxin reductase, Molecular modeling

## Abstract

**Background:**

The compounds 1,4-napthoquinone (1,4-NQ), bis-(2,4-dinitrophenyl)sulfide (2,4-DNPS), 4-nitrobenzothiadiazole (4-NBT), 3-dimethylaminopropiophenone (3-DAP) and menadione (MD) were tested for antimalarial activity against both chloroquine (CQ)-sensitive (D6) and chloroquine (CQ)-resistant (W2) strains of *Plasmodium falciparum* through an *in vitro* assay and also for analysis of non-covalent interactions with *P. falciparum* thioredoxin reductase (*Pf*TrxR) through *in silico* docking studies.

**Results:**

The inhibitors of *Pf*TrxR namely, 1,4-NQ, 4-NBT and MD displayed significant antimalarial activity with IC_50_ values of < 20 μM and toxicity against 3T3 cell line. 2,4-DNPS was only moderately active. *In silico* docking analysis of these compounds with *Pf*TrxR revealed that 2,4-DNPS, 4-NBT and MD interact non-covalently with the intersubunit region of the enzyme.

**Conclusions:**

In this study, tools for the identification of *Pf*TrxR inhibitors using phenotyphic screening and docking studies have been validated for their potential use for antimalarial drug discovery project.

## Background

Malaria, a tropical parasitic disease, continues to be the dominant cause of death in low-income countries especially in Africa and is considered to be one of the top three killers among communicable diseases [1]. Malaria caused by *Plasmodium falciparum* is considered to be the most deadly and also the one with highest rate of drug resistance [2]. Research investment in new and improved interventions will improve malaria cure, control, increase the cost-effectiveness of interventions and support efforts to eliminate malaria [3].

*P. falciparum* requires efficient antioxidant and redox systems to prevent damage caused by reactive oxygen species. In recent years, it has been shown that *P. falciparum* (*Pf*) possesses a functional low molecular weight thiol thioredoxin (Trx) system [4]. Thioredoxin reductase (TrxR) is an important enzyme of this redox system that helps the parasite to maintain an adequate intracellular redox environment during intraerythrocytic development and proliferation. This antioxidant enzyme (*Pf*TrxR) is essential for the survival of *Plasmodium* parasites for combating intraerythrocytic oxidative stress. Disruption of this enzyme is a feasible way to interfere with intraerythrocytic development and proliferation of the malaria parasites [5]. The current chemotherapy for malaria as recommended by WHO focuses on artemisinin-based combination therapies (ACTs) as the front line of treatment for malaria disease. The main drawbacks of combination therapies are high cost, adverse drug reactions and a high degree of pharmacokinetic mismatch between components leading to prolonged exposure of parasites to low doses of partner drug and its active metabolites which may facilitate development of resistant parasites [6].

Development of parasites’ resistance to the known antimalarials remains a major challenge for the effective management of malaria. Intensive drug discovery programs have aimed at developing new antimalarials or modifying current antimalarials to improve their efficacy and reduce evidence of resistance.

*In silico* molecular modeling methods, such as docking can aid in the drug discovery process by ascertaining the binding affinities of existing and hypothetical compounds towards *Pf*TrxR and the human isoform of this enzyme. Ideally, the simulations can also elucidate the origin behind the observed inhibition, as crystalline enzyme/inhibitor complexes of the thioredoxin protein for x-ray structure determination have not been reported. A comparison with other disulfide reductases including glutathione reductases reveals the most common inhibitor binding sites are at the active site and at the crystallographic 2-fold axis in the large cavity at the dimer interface. These sites can be exploited for structure-based inhibitor development. The dimer interface shows non-competitive or uncompetitive behavior and their interaction with the protein is purely non-covalent [7-10]. Docking calculations are well suited for exploration of this interface; however, the simulations are unable to reproduce covalent inhibitors that bind irreversibly at the active site. A combined experimental and computational effort may counterbalance this deficiency and provide an enhanced avenue for inhibitor development.

A comparison between the *h*TrxR and *Pf*TrxR structures shows that they have 46% sequence identity and overlay with an RMSD of 0.91 Å between the 374 monomer atom pairs. The most important difference that can be exploited for selective inhibition between the two enzymes is at the dimer interface. The interface in *Pf*TrxR is narrower than in *h*TrxR due to the presence of Tyr101 and His104 and can therefore host smaller molecules. Their counterparts in the human isoform are Gln72 and Leu75 and this difference can determine the chemical nature of suitable inhibitors [11]. The molecular surfaces of the parasite and the human enzymes also indicate that the charges on the cavity walls are different, with the *h*TrxR’s being more negatively charged compared to the *Pf*TrxR’s [11].

The current study is aimed to employ the combined approach of *in silico* molecular docking for identification of key interactions of *Pf*TrxR inhibitors to improve selectivity and phenotypic antimalarial assays for identification of activity against susceptible and drug-resistant *P. falciparum* blood stage cultures to assure the identification of specific *Pf*TrxR inhibitors as scaffolds for lead optimization.

## Results and discussion

**Figure 1:**
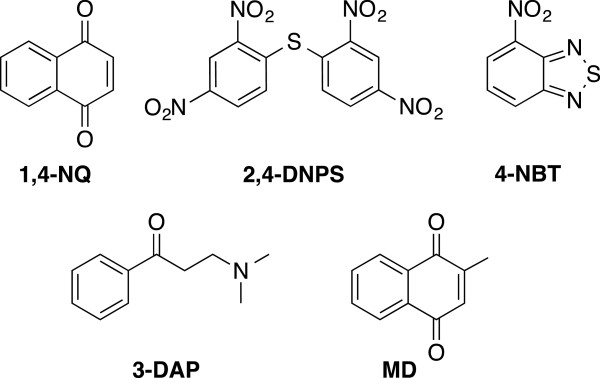
**The five *Pf*** **TrxR inhibitors.**

The *in vitro* antimalarial activity of the five known inhibitors of *Pf*TrxR (1,4-NQ, 2,4-DNPS, 4-NBT, 3-DAP, MD) [12,13] (Figure 1; Table 1) was evaluated against both CQ-sensitive (D6 clone) and CQ resistant (W2 clone) strains of *P. falciparum*, while cell cytotoxicity was determined against 3T3 cells (Table 1) using the procedure described earlier. The compounds 1,4-NQ and 4-NBT were found to be the most active against the two strains of *P. falciparum*, MD and 2,4-DNPS were moderately active, and 3-DAP was inactive. In terms of antiplasmodial activity against the W2 strain, 1,4-NQ and 4-NBT showed IC_50_ value of < 20 μM. The low correlation between the high *Pf*TrxR inhibitory activity and moderate antiplasmodial activity of 2,4-DNPS could be explained by an inability to penetrate the cell membranes. Accordingly, 2,4-DNPS is predicted to have poor Caco-2 and MDCK cell line permeability. Table 2 gives the computed octanol/water partition coefficient (logP), solubility in water (logS), polar surface area, and apparent Caco-2 and MDCK permeability for all compounds given in Figure 1, the enol form of 3-DAP, and CQ.

**Table 1:** ***Pf*** **TrxR inhibitory and antiplasmodial activities of tested compounds**

**Test compounds**	***Pf*** **TrxR IC**_**50 **_(μM)*	***Pf *** **(D6) CQ sensitive IC**_**50**_(μM)**	**SI D6**	***Pf *** **(W2) CQ resistance IC**_**50**_(μM)**	**SI W2**	**3T3 IC**_**50**_**(μM)**
1,4-NQ	0.75	8.9 ± 2.3	4.6	16.7 ± 3.7	2.4	38.5 ± 0.76
2,4-DNPS	0.5	91.2 ± 11.3	0.8	72.3 ± 11.3	1.0	79 ± 3.51
4-NBT	2	8.3 ± 2.1	10	9.8 ± 1.9	8	80 ± 1.15
3-DAP	15.4	>100	>1	>100	>1	>100
MD	1.6	18.5 ± 1.9	3.8	28.3 ± 5.6	2.5	70.5 ± 3.69
CQ		0.055 ± 0.006		0.440 ± 0.045		NC

*IC_50_ values, preparation of *Pf*TrxR and optimal experimental conditions for the *Pf*TrxR functional assay were reported in Andricopulo et al., 2006 [12]; Charvet et al., 2003 [13]; Munigunti et al., 2012 [14].

**Values are mean ± S.D. of triplicate observations; SI: Selectivity index. NC: no cytotoxicity up to concentration much higher than the concentration responsible for its antiplasmodial activity.

**Table 2:** Predicted physico-chemical properties of the compounds

**Molecule**	**log P octanol/water (M)**	**log S aqueous solubility (M)**	**Polar surface area (PSA)**	**Apparent caco-2 permeability (nm/sec)**	**Apparent MDCK permeability (nm/sec)**
1,4 NQ	0.486	−0.764	57.199	954	470
2,4 DNPS	1.406	−3.444	177.135	5	2
4-NBT	0.746	−1.414	75.626	382	385
3-DAP	1.249	−0.313	32.353	819	441
3-DAP-enol	1.800	−1.170	25.296	842	454
MD	0.880	−1.312	55.456	1225	616
CQ	4.276	−3.585	24	1364	1862
Range 95% of drugs	(−2.0 / 6.5)	(−6.5 / 0.5)	(7.0 / 330.0)	(<25 poor, >500 great)	(<25 poor, >500 great)

The lack of antiplasmodial activity of 3-DAP, a Mannich base, may be due to (i) non-specific alkylation of cellular thiol groups, and also (ii) due to the absence of active transport to red blood cells and parasites. The correlation between inhibition of *Pf*TrxR in the enzyme inhibition assays and antiplasmodial activity in cell culture allows for a better evaluation of biological activities of inhibitor compounds. The active compounds namely, 1,4-NQ, 2,4-DNPS, 4-NBT and MD showed more toxicity than 3-DAP against the 3T3 cell line. The 3T3 cells are epithelial cells that reflect toxicity against proliferating mammalian cells.

**Figure 2:**
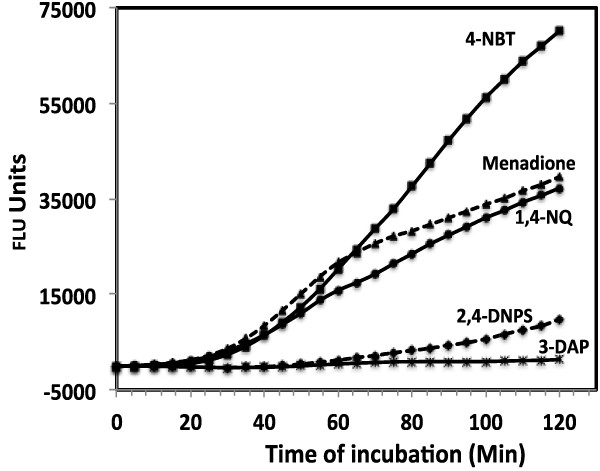
**Formation of reactive oxygen species (ROS), as indicated by increase in fluorescence.** DCFDA loaded human erythrocytes by five *Pf*TrxR inhibitors.

In order to test the five *Pf*TrxR inhibitors for their ability to induce signs of oxidative stress by accelerated generation and accumulation of reactive oxygen intermediates (superoxide radical, hydroxyl radical and hydrogen peroxide) [15] the intraerythrocytic formation of ROS was monitored in real-time for 120 min with 2′7′-dichlorofluorescein diacetate (DCFDA), a fluorescent ROS probe [16]. Among the compounds tested 4-NBT, MD and 1,4-NQ caused a significant increase in oxidative stress (Figure 2). Whereas, 3-DAP and 2,4-DPNS did not cause the production of ROS. These results suggest that 4-NBT, MD and 1,4-NQ compromises the capability of erythrocytes to scavenge reactive oxygen intermediates. The accumulated intra-erythrocytic oxidative stress by these compounds may be responsible for the inhibition of *h*TrxR enzyme. The erythrocytes, our target cells, have higher capacity to produce oxidative stress than 3T3 cell line used for cytotoxicity assessment.

The 1,4-NQ chemical features and the ability to generate ^·^OH suggest the proficiency in altering intracellular redox status [17]. The antimalarial naphthoquinones (1,4-NQ and MD) are believed to perturb the major redox equilibria of the targeted *P. falciparum* infected red blood cells, which might be removed by macrophages. This perturbation results in development arrest and death of the malaria parasite at the trophozoite stage [18].

Since these compounds were active against *Pf*TrxR as well, molecular docking was used to study their interactions with *Pf*TrxR to gain further insight into the mode of interaction for these molecules. MD [19], DNPS and 4-NBT [20] have been proposed to bind at the intersubunit region in *Pf*TrxR’s. However, 1,4-NQ and 3-DAP bind to the reductase covalently precluding the use of docking calculations. For example, 1,4-NQ is an inhibitor of TrxR that behaves as a subversive substrate [19]. The compound 3-DAP inactivates TrxR by alkylating the C-terminal redox active catalytic Cys-Cys pair. This is achieved by the formation of a reactive α, β-unsaturated ketone intermediate after it undergoes deamination in solution [13]. Therefore 3-DAP acts an alkylator. The calculations predict the same activity trend observed in the experimental IC_50_ values for the non-covalent inhibitors, 2,4-DNPS, MD, and 4-NBT in *Pf*TrxR (Table 3).

**Table 3:** **Comparison between computed binding affinities at the dimer interface in *Pf*** **TrxR and experimental IC**_**50 **_**values**

**Molecule**	**Computed binding affinity (kcal/mol)**	**Exptl. *Pf*** **TrxR IC**_**50**_**(μM)**
2,4-DNPS	−8.4	0.5
MD	−7.9	1.6
4-NBT	−6.0	2

**Figure 3:**
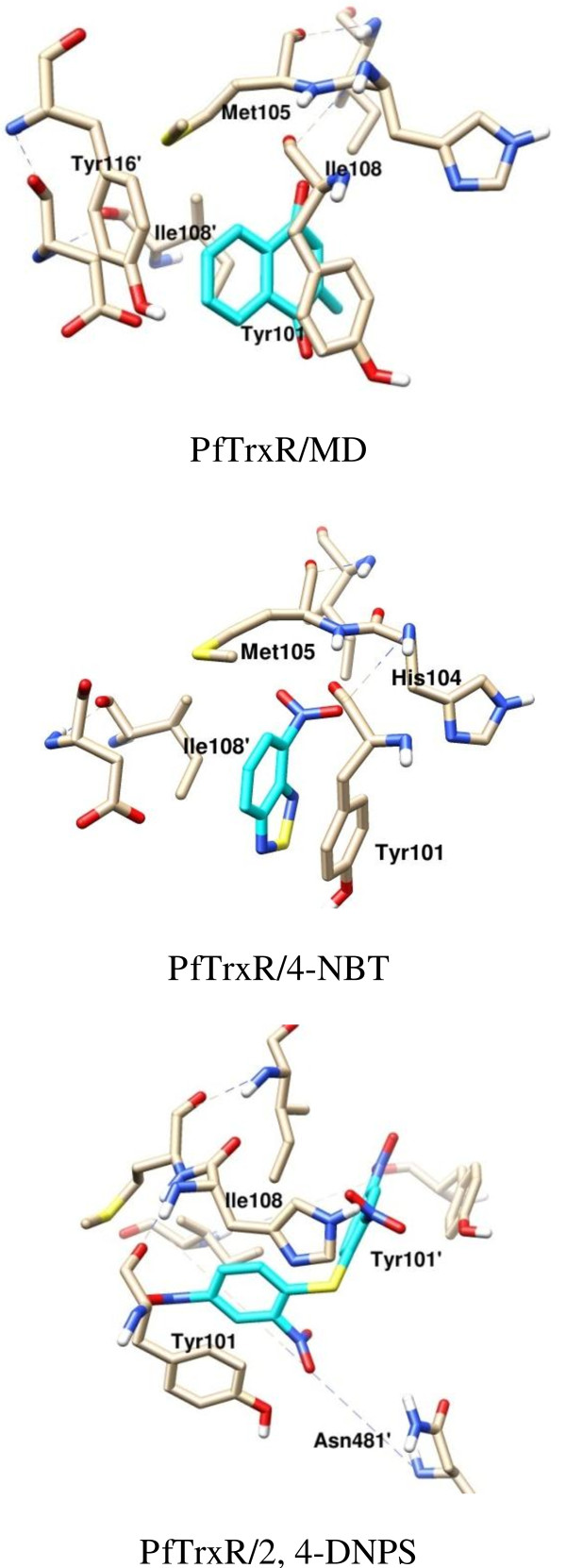
**The predicted binding poses for the inhibitors showing the main interactions with the dimer interface residues.***Pf*TrxR/MD, *Pf*TrxR/4-NBT, and *Pf*TrxR/2, 4-DNPS complexes.

For the *Pf*TrxR/MD complex, pi stacking interactions are predicted to form between the inhibitor’s phenyl ring and Tyr101 side chain ring. The backbone nitrogen of Met105 is in close proximity to the carbonyl group of MD; however, the predicted angle between N-H and O of 85° impedes hydrogen bonding. The molecule further forms hydrophobic interactions with the phenyl ring of Tyr116′ and the side chains of Ile108 from both subunits. Similar to MD, 4-NBT’s phenyl ring also has pi-pi stacking with Tyr101’s phenyl ring, but forms hydrophobic interactions only with Ile108 from subunit B in the large cavity. The nitro group causes the molecule to twist subtly compared to MD in order to better interact with the electrostatic surface created by the peptide bond between His104 and Met105’s and sulfur (Figure 3). Compared to the size of the cavity, MD and 4-NBT are small molecules and do not fully interact with most of the residues lining the wall of the dimer interface.

2,4-DNPS forms the only electrostatic interaction at a distance of 3.9 Å with Asn481′. Pi stacking interactions are formed between one of the inhibitor’s phenyl rings and Tyr101 side chain ring with the other phenyl ring of the molecule forming a parallel displaced pi stacking interaction with Tyr101′ (subunit B). As with MD and 4-NBT, the side chains of Ile108 from both subunits form hydrophobic interactions with 2,4-DNPS. Most of the interactions the three molecules are forming with the proteins are with the intersecting helices between the two subunits of the enzymes. The experimental activities for 2,4-DNPS and 4-NBT show selectivity between the parasite and human isoform of thioredoxin reductase (Table 4). The experimental values for 2,4-DNPS show an 8-fold selectivity for *Pf*TrxR, whereas 4-NBT has a 25-fold selectivity. While the docking simulations correctly predicted binding trends, limitations in the method, including potentially inaccurate scoring functions, the use of rigid proteins, and a lack of solvation could have contributed to its inability to reproduce the large differences observed in the IC_50_ values.

**Table 4:** **Comparison between computed binding affinities at the dimer interface and experimental IC**_**50 **_**values in *Pf*** **TrxR and *h*** **TrxR**

**Molecule**		***Pf*** **TrxR**	***h*** **TrxR**
2,4-DNPS	Exptl. IC_50_(μM)	0.5	4
	Calc. binding affinity (kcal/mol)	−8.4	−8.1
4-NBT	Exptl. IC_50_(μM)	2	50
	Calc. binding affinity (kcal/mol)	−6.0	−5.7

**Figure 4:**
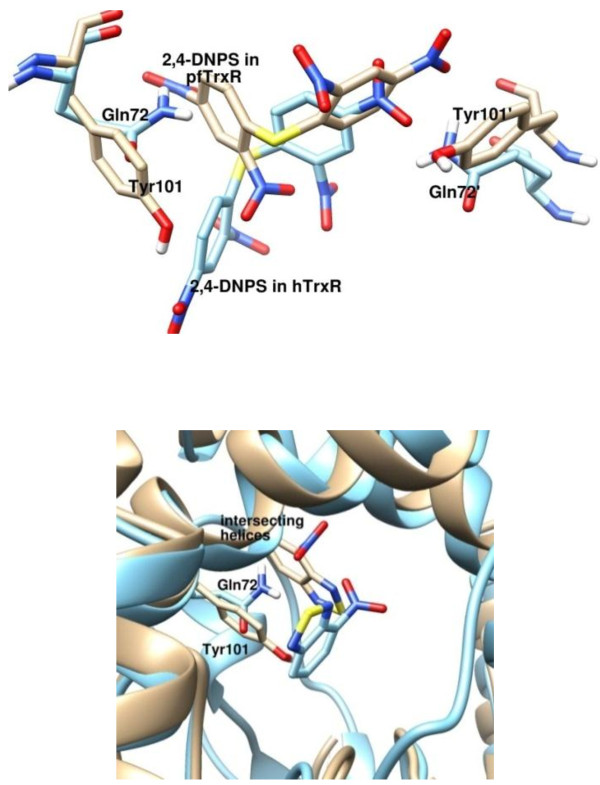
**The docking pose differences of 2,4-DNPS and 4-NBT between the *Pf*- (brown) and *h*-TrxR (blue).** The docked structures point to the difference in conformation between Try101 (*pf*TrxR) and Glu72 (*h*TrxR) which is proposed to have significant contribution to the observed experimental selectivity.

The docked poses, however, have considerable differences within the cavity, which could point to the observed selectivity (Figure 4). The presence of Tyr101 in *Pf*TrxR enables 2,4-DNPS to form a favorable pi stacking interaction with the phenyl ring of the molecule, whereas its counterpart in *h*TrxR is a Gln72 that orients the molecule to avoid steric clashes. This results in the second ring of the molecule forming a parallel displaced pi stacking interaction with Tyr101′, whereas a hydrogen bond between the nitro group and Gln72’ in the *h*TrxR is realized. The effect of this substitution on 4-NBT seems to be the fact that the presence of Gln72 pushes the molecule deep into the large cavity precluding the interaction with the residues of the intersecting helices between the subunits. 1,4-NQ and 4-NBT can be considered to be attractive leads for further optimization as these compounds display good *Pf*TrxR inhibitory and antiplasmodial activity.

**Figure 5:**
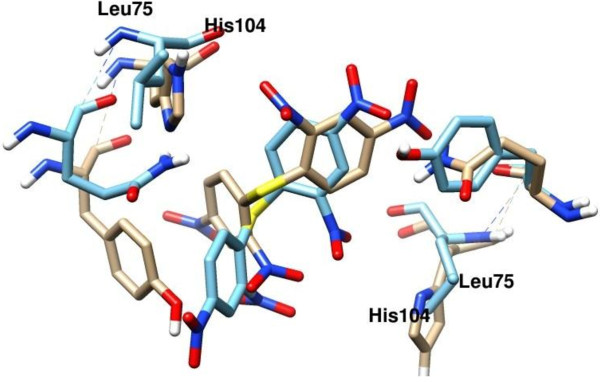
**The predicted binding mode of 2,4-DNPS in the *Pf*** **TrxR (beige) and *h*** **TrxR (blue) showing the position of the residues Try101 (*Pf*** **TrxR) and Glu72 (*h*** **TrxR).**

A thorough examination of the residues making any form of interaction with the small molecules showed that no other, including His104 (*Pf*TrxR) and its counterpart in *h*TrxR (Leu75), influences the differences in binding between the parasite and human isoform. Figure 5 shows 2,4-DNPS docked in both proteins especially showing the positions of the His104 and Leu75 as an example.

## Conclusions

In this study, tools for the identification of *Pf*TrxR inhibitors using phenotypic screening and docking studies have been validated for their potential use for antimalarial drug discovery project.

### Experimental

#### Chemicals and enzymes

Deionized water generated by a Milli-Q water system (Millipore, MA) was used in the experiments. All reagents were purchased from Sigma–Aldrich.

### Biological assays

#### Antimalarial assay

Briefly, antimalarial activity of the compounds were determined *in vitro* on chloroquine sensitive (D6, Sierra Leone) and resistant (W2, IndoChina) strains of *P. falciparum*. The 96-well microplate assay is based on the effect of the compounds on growth of asynchronous cultures of *P. falciparum*, as determined by the fluorometric SYBR green assay [21].

#### Cytotoxicity assay

Cytotoxicity in terms of cell viability was evaluated using 3T3 cells by AlamarBlue assay [22]. This assay was conducted on compounds designated as active in the *Pf*TrxR functional assay and the antimalarial phenotypic screening.

#### ROS assay

Accelerated generation and accumulation of reactive oxygen intermediates (superoxide radical, hydroxyl radical and hydrogen peroxide) are mainly responsible for oxidative stress [15]. The intraerythrocytic formation of ROS was monitored in real-time with 2′7′-dichlorofluorescein diacetate (DCFDA), a fluorescent ROS probe [16]. Human erythrocytes collected in citrate phosphate anticoagulant were used. The erythrocytes were washed twice with 0.9% saline and suspended in PBSG at a hematocrit of 10%. A 60 mM stock of DCFDA was prepared in DMSO and added to the erythrocytes suspension in PBSG (10% hematocrit) to obtain the final concentration of 600 μM. Erythrocytes suspension containing 600 μM of DCFDA was incubated at 37°C for 20 min and centrifuged at 1000 *g* for 5 min. The pellet of DCFDA loaded erythrocytes was suspended in PBSG to 50% hematocrit and used for kinetic ROS formation assay. The assay was directly set up in a clear flat-bottom 96 well microplate. The reaction mixture contained 40 μl of DCFDA loaded erythrocytes, the test compounds (50 μM)) and potassium phosphate buffer (100 mM, pH 7.4), to make up the final volume to 200 μl. The controls without drug were also set up simultaneously. Each assay was set up at least in duplicate. The plate was immediately placed in a microplate reader programmed to kinetic measurement of fluorescence (excitation 488 nm and emission 535 nm) for 2 hours with 5 min time intervals.

### Computational studies

#### Computational methods

AutoDock Vina [22] was used to dock inhibitors to the respective targets. Initial Cartesian coordinates for the protein-ligand structures were derived from reported crystal structures of *h*TrxR (PDB ID: 3QFA) [23] and *Pf*TrxR (PDB ID: 4B1B) [11]. The protein targets were prepared for molecular docking simulation by removing water molecules and bound ligands. AutoDockTools (ADT) [24] was used to prepare the docking simulations whereas Chimera was used to analyze the docking poses. All ligands were constructed using PyMol [25] with subsequent geometry optimizations carried out using the semi-empirical method PDDG/PM3 [20,26,27]. Polar hydrogens were added. ADME properties logP, logS, polar surface area, and apparent Caco-2 permeability for each ligand were computed using QikProp [28,29]. Conjugate gradient minimizations of the systems were performed using GROMACS [30]. A grid was centered on the catalytic active site region and included all amino acid residues within a box size set at x = y = z = 20 Å.

#### AutoDock Vina details

Standard flexible protocols of AutoDock Vina using the Iterated Local Search global optimizer [31] algorithm were used to evaluate the binding affinities of the molecules and interactions with the receptors. All ligands and docking site residues, as defined by the box size used for the receptors, were set to be rotatable. Calculations were carried out with the exhaustiveness of the global search set to 100, number of generated binding modes set to 20 and maximum energy difference between the best and the worst binding modes set to 5. Following completion of the docking search, the final compound pose was located by evaluation of AutoDock Vina’s empirical scoring function where the conformation with the lowest docked energy value was chosen as the best.

## Competing interests

All the authors declare that they have no competing interests.

## Authors’ contributions

AIC supervised all the research work. RM carried out the *Pf*TrxR inhibitory assays and wrote the manuscript, which is part of his Ph.D. thesis. SG performed the docking experiments and helped writing the section. OA supervised the experiments and results of docking experiments. RS conducted the antimalarial and cytotoxicity assays. BT supervised the experiments and results of the antimalarial and cytotoxicity assays. All authors read and approved the manuscript.
